# Sequencing of chemotherapy in total neoadjuvant treatment for rectal cancer does not predict radiation-induced lymphopenia

**DOI:** 10.2478/raon-2025-0034

**Published:** 2025-06-16

**Authors:** Miha Orazem, Vaneja Velenik, Alojz Ihan

**Affiliations:** 1 Division of Radiotherapy, Institute of Oncology Ljubljana, Ljubljana, Slovenia; 2 Faculty of Medicine, University of Ljubljana, Ljubljana, Slovenia; 3 Institute of Microbiology and Immunology, Faculty of Medicine, University of Ljubljana, Ljubljana, Slovenia

**Keywords:** radiation-induced lymphopenia, rectal cancer, total neoadjuvant treatment

## Abstract

**Background:**

Radiation-induced lymphopenia (RIL) is associated with an increased risk of death in solid tumors, including rectal cancer. The aim of this study was to determine whether the sequencing of chemotherapy in total neoadjuvant treatment (TNT) for rectal cancer predicts the development of RIL.

**Patients and methods:**

We analyzed acute hematologic toxicity data from 53 patients who underwent TNT for locally or locoregionally advanced rectal cancer between July 2022 and April 2023. Twenty-eight patients received induction chemotherapy with capecitabine and oxaliplatin [CAPOX], and 25 received consolidation chemotherapy (6 cycles of CAPOX in both groups). The chemoradiation protocol consisted of Volumetric Modulated Arc Therapy with Simultaneous Integrated Boost Radiotherapy (VMAT-SIB RT) up to 48.4 Gy in 22 fractions, concomitantly with capecitabine twice a day (*lat. bis in die*, BID). The Mann-Whitney U test was performed to compare RIL between the two patient groups. Pelvic bone marrow was contoured as a non-limiting organ-at-risk to assess the received dose, and binary logistic regression was used to determine whether RIL depends on V_5Gy_~V_42Gy_ or the planning target volume (PTV) size.

**Results:**

Thirty-four patients (64.2%) developed RIL of any grade, which was not significantly associated with either the induction or consolidation chemotherapy TNT regimen (Wald = 3.159, p = 0.076). No significant differences were found in neutrophil counts or the neutrophil-to-lymphocyte ratio. In the logistic regression model predicting the likelihood of RIL, two variables were statistically significant: V_10Gy_ (Wald = 4.366, p = 0.037) and V_30Gy_ (Wald = 6.084, p = 0.014). These results indicate that V_10Gy_< 71% and V_30Gy_< 26.6% may reduce the likelihood of developing RIL.

**Conclusions:**

In our study, the sequencing of chemotherapy in TNT for rectal cancer did not predict the development of RIL. However, the incidence of RIL may be reduced by applying RT dosimetric constraints to the pelvic bone marrow.

## Introduction

Radiation-induced lymphopenia (RIL) has been associated with a poorer prognosis in patients with solid tumors.^1–3^ It develops due to the direct cytotoxic effects of radiation on lymphocytes as a result of circulating blood pool exposure, as well as the impact on lymphoid tissues and bone marrow, which depends on the size and location of the treatment field.^4^ The severity of RIL is additionally influenced by concurrent cytotoxic or immunosuppressive systemic therapy. Patients with persistently low absolute lymphocyte count, irrespectively of grade are at increased risk for tumor progression and opportunistic infections due to compromised adaptive immunity.^5,6^

Total neoadjuvant treatment (TNT), which includes chemoradiation combined with either induction or consolidation chemotherapy, has become the standard of care for locally and regionally advanced rectal cancer with high risk of recurrence.^7^ This approach not only improves disease control and survival outcomes but also serves as a strategy to avoid surgery and promote organ preservation.^8,9^ However, little is known about the impact of chemotherapy sequencing on RIL. Since consolidation chemotherapy appears to be the preferred option for organ preservation, it is essential to examine how these two different sequencing strategies influence RIL and their potential implications for treatment outcomes.

## Patients and methods

### Patient selection and treatment protocol

We analyzed acute hematologic toxicity data from 53 patients who underwent TNT for locally or locoregionally advanced rectal cancer between July 2022 and April 2023 (IKONA trial, NCT05054959). Among them, 28 patients received induction chemotherapy, while 25 received consolidation chemotherapy. Induction regimen consisted of 4 cycles of capecitabine (1000 mg/m^2^ twice a day [BID], orally) administered from the first to the 14th day of each cycle, along with a single intravenous dose of oxaliplatin (130 mg/m^2^) on the first day of each cycle (CAPOX), followed by chemoradiation and afterwards an additional 2 cycles of CAPOX. The consolidation regimen consisted of six cycles of CAPOX, followed by chemoradiation.

**FIGURE 1.**
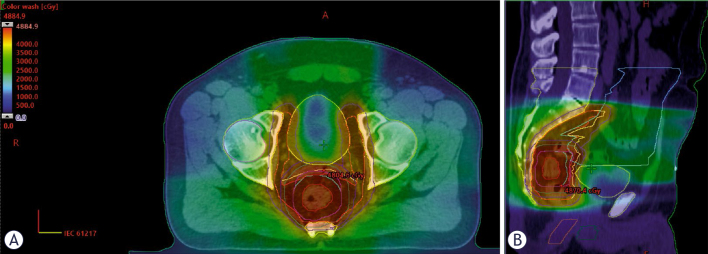
Dose distribution of volumetric modulated arc therapy with simultaneous integrated boost in a representative patient with locally advanced rectal cancer undergoing total neoadjuvant treatment. Bone marrow contour is in yellow. **(A)** Axial view. **(B)** Sagittal view.

The chemoradiation protocol included Volumetric Modulated Arc Therapy with Simultaneous Integrated Boost Radiotherapy (VMAT-SIB RT). Pelvic lymph nodes received 41.8 Gy in 22 fractions, with a Simultaneous Integrated Boost (SIB) to the gross tumor volume (GTV) up to 46.2 Gy, or 48.4 Gy in the case of T4 tumors (Figure 1). RT was administered concomitantly with capecitabine (825 mg/m^2^ BID), which was given continuously from the first to the last day of radiotherapy, including weekends.

### Data collection

Absolute lymphocyte and neutrophil counts were obtained from the hospital information system as part of routine peripheral blood work, collected at both the beginning and end of treatment. The neutrophil-to-lymphocyte ratio (NLR) was calculated from these values. Toxicity was graded according to the Common Terminology Criteria for Adverse Events (CTCAE), version 5.0 (grade 1 < lower level of normal - 0.8 × 10^9^/L; grade 2 < 0.8–0.5 × 10^9^/L; grade 3 < 0.5–0.2 × 10^9^/L; grade 4 < 0.2 × 10^9^/L).

In the planning CT scans, pelvic bone marrow was contoured as a non-limiting organ-at-risk to assess the received radiation dose and its correlation with the planning target volume (PTV) size. Dosimetric data for bone marrow volumes receiving 5 to 42 Gy (V_5Gy_–V_42Gy_) were extracted from dose-volume histograms using the Varian Eclipse treatment planning system, version 15.1.

### Statistical analysis

Statistical analysis was conducted using SPSS software (Statistical Package for the Social Sciences, version 29.0, IBM Corp., Armonk, NY, USA). The Mann-Whitney U test was used to compare RIL between the two patient groups, with a p-value of less than 0.05 considered statistically significant. Additionally, binary logistic regression was performed to identify factors influencing the likelihood of RIL development.

The study was approved by the National Medical Ethics Committee of the Republic of Slovenia (Ref. No. 0120-214/2021).

## Results

### Lymphopenia

Patient and dosimetric characteristics are shown in Table 1. Thirty-four patients (64.2%) developed RIL of any grade, which was not significantly associated with either the induction or consolidation chemotherapy TNT regimen (Wald = 3.159, p = 0.076). Grade 1, 2, and 3 lymphopenia were developed by 20, 12, and 2 patients, respectively, while no Grade 4 lymphopenia was recorded.

**TABLE 1.** Patient, lymphocyte count and dosimetric characteristics

	Induction chemotherapy	Consolidation chemotherapy
Number of patients	28	25
Sex	25 M, 3 F	20 M, 5 F
Mean age at the start of treatment (years)	59.1 (37-76)	54.3 (33-72)
Mean absolute lymphocyte count at the start of treatment	2.13 × 10^9^/L (0.78 IQR)	1.95 × 10^9^/L (1.03 IQR)
Mean absolute lymphocyte count at the end of treatment	1.12 × 10^9^/L (0.52 IQR)	0.92 × 10^9^/L (0.37 IQR)
Mean change in lymphocyte count	-1.05 × 10^9^/L (0.77 IQR)	-1.10 × 10^9^/L (1.02 IQR)
Number of patients with RIL	15 (53.6%)	19 (76.0%)
Mean PTV size	1273 mL (299 IQR)	1259 mL (378 IQR)
Mean pelvic bone marrow volume	1767 mL (304 IQR)	1693 mL (362 IQR)
Mean dose to pelvic bone marrow	20.38 Gy (3.04 IQR)	20.43 Gy (2.16 IQR)
V_5Gy_	80.23% (8.48 IQR)	80.26% (8.75 IQR)
V_10Gy_	71.34% (10.0 IQR)	69.43% (11.1 IQR)
V_20Gy_	50.95% (9.1 IQR)	51.06% (8.6 IQR)
V_30Gy_	26.65% (8.2 IQR)	26.56% (6.9 IQR)
V_40Gy_	10.25% (3.6 IQR)	10.10% (2.9 IQR)
V_42Gy_	5.47 % (2.1 IQR)	4.90% (2.5 IQR)

1 F = female, Gy = Gray, IQR = interquartile range, L = liter, M = male, PTV = planning target volume, RIL = radiation-induced lymphopenia, TNT = total neoadjuvant treatment, V_x_ = volume of pelvic bone marrow that receives × Gy

In the logistic regression model predicting the likelihood of RIL, two variables were statistically significant: V_10Gy_ (Wald = 4.366, p = 0.037) and V_30Gy_ (Wald = 6.084, p = 0.014). These results indicate that V_10Gy_< 71% and V_30Gy_< 26.6% may reduce the likelihood of developing RIL. The size of PTV did not predict RIL.

### Neutropenia and NLR

No cases of neutropenia below 1.5 × 10^9^/L were recorded at the end of treatment. Pre- and post-treatment neutrophil-to-lymphocyte ratio (NLR) values are presented in Table 2. No significant differences were observed between the groups.

**TABLE 2.** Neutrophil-to-lymphocyte ratio (NLR) pre- and post-treatment

	Induction chemotherapy	Consolidation chemotherapy
Mean absolute neutrophil count at the start of treatment	5.15 × 10^9^/L (1.94 IQR)	5.79 × 10^9^/L (2.12 IQR)
Mean absolute neutrophil count at the end of treatment	2.79 × 10^9^/L (0.88 IQR)	3.03 × 10^9^/L (1.53 IQR)
Mean change in neutrophil count	-2.35 × 10^9^/L (1.63 IQR)	-2.76 × 10^9^/L (2.88 IQR)
Mean pre-treatment NLR	2.76 (1.18 IQR)	3.27 (2.33 IQR)
Mean post-treatment NLR	2.83 (1.64 IQR)	3.73 (1.49 IQR)
Mean change NLR	+0.07 (1.49 IQR)	+0.46 (2.44 IQR)

1 IQR = interquartile range, NLR = neutrophil-to-lymphocyte ratio

## Discussion

This is the first study, to our knowledge, to explore the relationship between the sequencing of chemotherapy within TNT for rectal cancer and the development of RIL. Our data indicate no significant association between induction or consolidation chemotherapy and the risk of RIL. This finding is particularly important because several studies have shown that starting with radiotherapy, followed by consolidation chemotherapy, increases the likelihood of a complete clinical response of the tumor and, consequently, the number of patients who can undergo a ‘watch-and-wait’ approach as an organ preservation strategy.^10,11^

The two most notable phase 2 randomized trials, CAO/ARO/AIO-12 and OPRA, that both compared induction and consolidation chemotherapy did not report lymphopenia rates, but acute hematologic toxicity described by neutropenia, febrile neutropenia and low platelets count. No differences were observed between groups.^12–15^ Lymphopenia as a side effect of radiotherapy in rectal cancer was recently assessed in a retrospective study comparing fractionation schedules.^16^ Lymphocyte count declines were greater in normofractionation groups (1.8 Gy or 2 Gy per fraction) than in hypofractionation groups (3.4 Gy or 5 Gy per fraction). In their analysis, V_30Gy_< 30% of bone marrow volume was associated with a reduced risk of RIL, which is consistent with our observation of V_30Gy_< 26.6%. Our other suggested constraint (V_10Gy_< 71%) appears to be slightly more conservative as studies exploring haematological toxicity in cervical cancer recommended cut-off values of V_10Gy_ in the 75–95% range.^17^

Our results should be interpreted solely in the context of acute toxicity outcomes, not the potential long-term impact on bone marrow function, which represents a limitation of this study. Research on patients with advanced oral cancer has shown that, unlike surgery, lymphopenia following radiotherapy can persist for at least one year after treatment completion.^18,19^ It would therefore be valuable to investigate whether recovery rates after the subacute toxicity period differ between the two TNT groups. This area of research could also prove valuable with the adoption of immunotherapy in this setting.^20^ For instance, in the preliminary results of the PKUCH 04 trial that incorporated PD-1 blockade in the TNT protocol, Grade 3 lymphopenia occurred in 24% of the patients.^21^

It should be noted that the term ‘radiation-induced’ may be potentially misleading, since lymphopenia can also be exacerbated by systemic treatment. A more appropriate general term might be ‘treatment-related lymphopenia.’^22^ However, our study also aimed to evaluate the influence of the radiation dose to the bone marrow, the organ at risk, which explains our deliberate choice to use the term radiation-induced lymphopenia (RIL).

We acknowledge that there is likely underreported variability in bone marrow contouring. Typically, whole pelvic bones, including the fifth lumbar vertebra (L5), are delineated as a surrogate for pelvic bone marrow.^23^ However, in cervical cancer, freehand contouring of low-density bone marrow regions has been shown to better predict higher-grade hematologic toxicity. In our study, we used whole pelvic bone contouring, currently more widely adopted technique in clinical practice.^24^

## Conclusions

In conclusion, our study provides novel insights into the relationship between TNT sequencing and RIL in rectal cancer, demonstrating no significant impact of induction versus consolidation chemotherapy on acute lymphopenia risk, alongside identifying bone marrow dose constraints (V_10Gy_ < 71%, V_30Gy_< 26.6%) that may mitigate this toxicity. These findings align with recent evidence on fractionation and dosimetry effects. Future studies should explore long-term bone marrow recovery in this setting, refine bone marrow contouring techniques, and assess the implications of RIL for the emerging immunotherapy era in organ preservation treatment strategies.

## References

[1] El Houat Y, Massard C, Quillien V, de Crevoisier R, Castelli J. (2022). Meta-analysis and critical review: association between radio-induced lymphopenia and overall survival in solid cancers. Adv Radiat Oncol.

[2] Duque-Santana V, López-Campos F, Martin-Martin M, Valero M, Zafra-Martín J, Couñago F (2023). Neutrophil-to-lymphocyte ratio and platelet-to-lymphocyte ratio as prognostic factors in locally advanced rectal cancer. Oncology.

[3] Pham TN, Coupey J, Thariat J, Valable S. (2025). Impact of circulating lymphocyte kinetics following radiotherapy on patient survival: a model-based meta-analysis. Comput Biol Med.

[4] Ellsworth SG. (2018). Field size effects on the risk and severity of treatment-induced lymphopenia in patients undergoing radiation therapy for solid tumors. Adv Radiat Oncol.

[5] Prades-Sagarra E, Yaromina A, Dubois LJ. (2024). Understanding the impact of radiation-induced lymphopenia: preclinical and clinical research perspectives. Clin Transl Radiat Oncol.

[6] Terrones-Campos C, Ledergerber B, Vogelius IR, Specht L, Helleberg M, Lundgren J. (2019). Lymphocyte count kinetics, factors associated with the end-of-radiation-therapy lymphocyte count, and risk of infection in patients with solid malignant tumors treated with curative-intent radiation therapy. Int J Radiat Oncol Biol Phys.

[7] Tuta M, Boc N, Brecelj E, Omejc M, Anderluh F, Ermenc AS (2019). Total neoadjuvant treatment of locally advanced rectal cancer with high risk factors in Slovenia. Radiol Oncol.

[8] Fokas E, Schlenska-Lange A, Polat B, Klautke G, Grabenbauer GG, Fietkau R (2022). Chemoradiotherapy plus induction or consolidation chemotherapy as total neoadjuvant therapy for patients with locally advanced rectal cancer: long-term results of the CAO/ARO/AIO-12 randomized clinical trial. JAMA Oncol.

[9] Verheij FS, Omer DM, Williams H, Lin ST, Qin LX, Buckley JT (2024). Longterm results of organ preservation in patients with rectal adenocarcinoma treated with total neoadjuvant therapy: the randomized phase II OPRA trial. J Clin Oncol.

[10] Bedrikovetski S, Traeger L, Seow W, Dudi-Venkata NN, Selva-Nayagam S, Penniment M (2024). oncological outcomes and response rate after total neoadjuvant therapy for locally advanced rectal cancer: a network metaanalysis comparing induction vs. consolidation chemotherapy vs. standard chemoradiation. Clin Colorectal Cancer.

[11] Fokas E, Williams H, Diefenhardt M, Lin S, Qin LX, Piso P (2024). Chemoradiotherapy plus induction or consolidation chemotherapy as total neoadjuvant therapy for locally advanced rectal cancer: pooled analysis of the CAO/ARO/AIO-12 and the OPRA randomized phase 2 trials. Eur J Cancer.

[12] Fokas E, Allgäuer M, Polat B, Klautke G, Grabenbauer GG, Fietkau R (2019). Randomized phase II trial of chemoradiotherapy plus induction or consolidation chemotherapy as total neoadjuvant therapy for locally advanced rectal cancer: CAO/ARO/AIO-12. J Clin Oncol.

[13] Garcia-Aguilar J, Patil S, Gollub MJ, Kim JK, Yuval JB, Thompson HM (2022). Organ preservation in patients with rectal adenocarcinoma treated with total neoadjuvant therapy. J Clin Oncol.

[14] Verheij FS, Omer DM, Lin ST, Yuval JB, Thompson HM, Kim JK (2024). Compliance and toxicity of total neoadjuvant therapy for rectal cancer: a secondary analysis of the OPRA Trial. Int J Radiat Oncol Biol Phys.

[15] Zimmermann M, Richter A, Weick S, Exner F, Mantel F, Diefenhardt M (2022). Acute toxicities of patients with locally advanced rectal cancer treated with intensified chemoradiotherapy within the CAO/ARO/AIO-12 trial: comparing conventional versus VMAT planning at a single center. Sci Rep.

[16] Nanos C, Koukourakis IM, Mulita A, Avgousti R, Kouloulias V, Zygogianni A (2024). Lymphopenia induced by different neoadjuvant chemo-radiotherapy schedules in patients with rectal cancer: bone marrow as an organ at risk. Curr Oncol.

[17] Corbeau A, Kuipers SC, de Boer SM, Horeweg N, Hoogeman MS, Godart J (2021). Correlations between bone marrow radiation dose and hematologic toxicity in locally advanced cervical cancer patients receiving chemoradiation with cisplatin: a systematic review. Radiother Oncol.

[18] Dovšak T, Ihan A, Didanovič V, Kansky A, Ihan Hren N. (2009). Influence of surgical treatment and radiotherapy of the advanced intraoral cancers on complete blood count, body mass index, liver enzymes and leukocyte CD64 expression. Radiol Oncol.

[19] Dovšak T, Ihan A, Didanovič V, Kansky A, Verdenik M, Hren NI. (2018). Effect of surgery and radiotherapy on complete blood count, lymphocyte subsets and inflammatory response in patients with advanced oral cancer. BMC Cancer.

[20] Zhu J, Lian J, Xu B, Pang X, Ji S, Zhao Y (2023). Neoadjuvant immunotherapy for colorectal cancer: right regimens, right patients, right directions?. Front Immunol.

[21] Li Y, Pan C, Gao Y, Zhang L, Ji D, Cui X (2024). Total neoadjuvant therapy with PD-1 blockade for high-risk proficient mismatch repair rectal cancer. JAMA Surg.

[22] Grossman SA, Ellsworth S, Campian J, Wild AT, Herman JM, Laheru D (2015). Survival in patients with severe lymphopenia following treatment with radiation and chemotherapy for newly diagnosed solid tumors. J Natl Compr Canc Netw.

[23] Huang W, Dang J, Li Y, Cui HX, Lu WL, Jiang QF. (2021). Effect of pelvic bone marrow sparing intensity modulated radiation therapy on acute hematologic toxicity in rectal cancer patients undergoing chemo-radiotherapy. Front Oncol.

[24] Mahantshetty U, Krishnatry R, Chaudhari S, Kanaujia A, Engineer R, Chopra S (2012). Comparison of 2 contouring methods of bone marrow on CT and correlation with hematological toxicities in non-bone marrowsparing pelvic intensity-modulated radiotherapy with concurrent cisplatin for cervical cancer. Int J Gynecol Cancer.

